# Xp11.22 duplications in four unrelated Chinese families: delineating the genotype-phenotype relationship for *HSD17B10* and *FGD1*

**DOI:** 10.1186/s12920-020-0728-8

**Published:** 2020-05-07

**Authors:** Qingming Wang, Pengliang Chen, Jianxin Liu, Jiwu Lou, Yanhui Liu, Haiming Yuan

**Affiliations:** 1 Dongguan Maternal and Child Health Care Hospital, Dongguan, 523120 China; 2 Dongguan Institute of Reproductive and Genetic Research, Dongguan, 523120 China

**Keywords:** Xp11.22 duplication, Intellectual disability, *HUWE1*, *HSD17B10*, *FGD1*

## Abstract

**Background:**

Xp11.22 duplications have been reported to contribute to nonsyndromic intellectual disability (ID). The *HUWE1* gene has been identified in all male Xp11.22 duplication patients and is associated with nonsyndromic ID. Currently, few Xp11.22 duplication cases have been reported in the Chinese population, with limited knowledge regarding the role of other genes in this interval.

**Case presentation:**

We investigated four unrelated Chinese male Xp11.22 duplication patients, performed a comprehensive clinical evaluation for the patients and discussed the role of other genes in this interval. All patients presented with similar clinical features, including ID, speech impairments and motor delay, which were mostly consistent with those of the Xp11.22 duplication described previously. We searched and compared all cases and noted that one of the probands (Family 1) and DECIPHER case 263,219, who carried small overlapping duplications at Xp11.22 that only covered the entire *HSD17B10* gene*,* also suffered from ID, suggesting the important role of *HSD17B10* in this interval. Furthermore, three patients (two probands in Families 3 and 4 and DECIPHER case 249,490) had strikingly similar hypogonadism phenotypes, including micropenis, small testes and cryptorchidism, which have not been previously described in Xp11.22 duplication patients. Interestingly, the *FGD1* gene was duplicated only in these three patients. Sufficient evidence has suggested that haploinsufficiency of the *FGD1* gene causes Aarskog-Scott syndrome, which is characterized by hypogonadism and other abnormalities. Given that, we are the first group to propose that *FGD1* may be a potential dosage-sensitive gene responsible for the hypogonadism observed in our patients.

**Conclusion:**

We reported novel genotypes and phenotypes in Chinese male Xp11.22 duplication patients, and the *HSD17B10* and *FGD1* genes may be involved.

## Background

Xp11.23p11.22 duplication is a recurrent copy number variant (CNV) mediated by the non-allelic homologous recombination (NAHR) between flanking low copy repeats (LCRs), which is a gene-rich region implicated in neurodevelopmental abnormalities, including ID, severe language delay, autistic behaviors, epilepsy and early onset of puberty [1–6]. Within this region, the nonrecurrent duplication at Xp11.22 has inconsistent breakpoints and is a relatively gene-poor region associated with nonsyndromic ID. To date, 18 unrelated families with overlapping duplications at Xp11.22 have been described, and most patients share similar clinical features, including ID, delayed motor development and delayed language [6–10]. Comparing these duplicated segments, the *HUWE1* gene, coding the E3 ubiquitin protein ligase, turned out to be duplicated in all previously described patients. Therefore, an increased dosage of *HUWE1* is believed to be responsible for nonsyndromic ID [6–10]. To date, little is known about the clinical consequences of other genes involved in this interval, and the genotype-phenotype relationship is still limited. Here, we present four additional, unrelated Chinese families with male patients suffering from mild to moderate ID and developmental delay, all of whom harbor overlapping duplications at Xp11.22. The proband (Family 1) and DECIPHER case 263,219 have ID and carry small Xp11.22 duplications partially encompassing *HUWE1*, suggesting the involvement of other ID genes in this interval. It is also interesting that two of our probands (Families 3 and 4) and DECIPHER case 249,490 show a similar hypogonadism phenotype that has not been described in previously reported patients with Xp11.22 duplication. In this study, we further explored the genotype-phenotype associations and the potential genes involved in the process.

## Case presentation

### Family 1

**Fig. 1:**
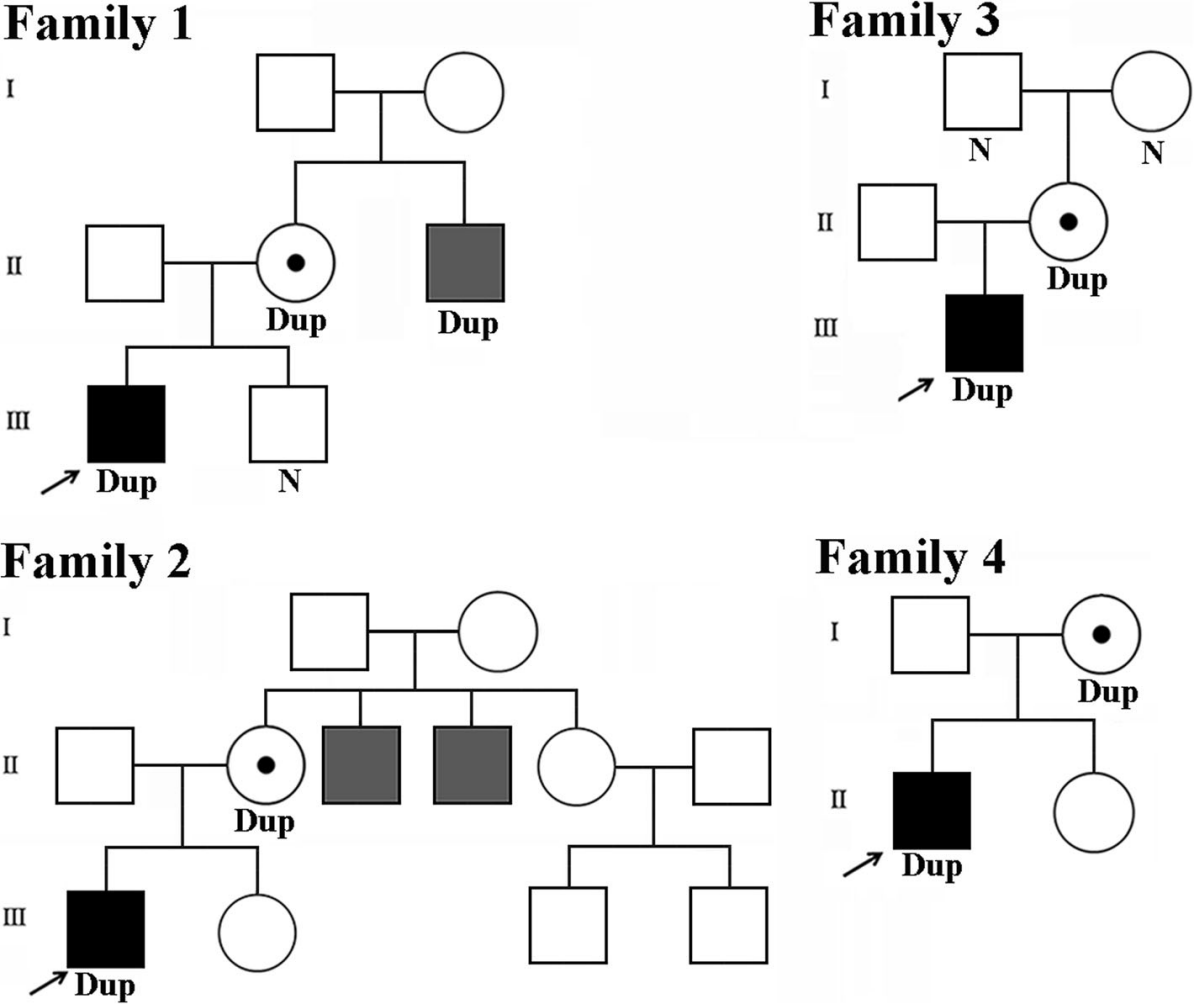
Pedigrees of four unrelated Chinese families with a nonrecurrent duplication at Xp11.22. All tested individuals are marked with Dup if the duplication is found in this individual or N if the duplication is not present, and the unmarked shows that the test is not available. The black-filled symbol represents the proband, who is also marked with an arrow, the gray-filled symbols represent affected individuals who suffered from intellectual disability, and the unfilled symbols represent apparently unaffected individuals

The proband was the first male child of a non-consanguineous couple. His 2-year-old younger brother was unaffected. His only maternal uncle had ID and speech impairments (Fig. 1). The proband was born at 38 weeks of gestation with a weight, height, and head circumference well within the normal ranges. His weight was 11.5 kg (32%), height 86 cm (44.6%), and head circumference 48 cm (46%) on physical examination at 1 year 10 months because of developmental delay. He had no language development and suffered from cognitive impairments. Motor development was significantly delayed: he raised his head at 8 months, sat alone at 1 year and could not independently stand.

On last physical examination at the age of 4 years, growth parameters were normal, and no dysmorphic facial features were recognized. He presented with remarkable absence of speech and mild ID with an intelligence quotient of 70 by the Wechsler Preschool and Primary Scale of Intelligence (WPPSI-III). He could walk independently, but his gait was unstable, and he fell frequently. Brain magnetic resonance imaging was normal.

### Family 2

The male proband was the second child of healthy, unrelated parents. The family history was obviously remarkable. His elderly sister was healthy. Both maternal uncles suffered from ID and limited speech. The unaffected maternal aunt had two healthy sons (Fig. 1). The proband’s mother was treated with progesterone during pregnancy due to signs of spontaneous abortion. The boy was born at 38 weeks gestational age by spontaneous vaginal delivery. The birth weight was 3 kg (20.3%) and length 50 cm (40.9%). No feeding problems were noted after birth. At the age of 9 months, he was referred to the clinic due to developmental delay.

Molecular genetic testing was performed for the boy at the age of 4 years and 4 months. His growth development was within the normal range: weight 17 kg (43.1%), height 106 cm (45.7%), and head circumference 50.5 cm (49.9%). He had delayed motor development: he raised his head at 5 months, sat alone at 10 months and walked without assistance at 1.5 years. His language development was remarkably delayed with only two-word formed sentences. He presented with mild ID and attention deficit hyperactivity disorder (ADHD).

At age 7, normal growth development and the absence of distinctive facial features were confirmed. He was diagnosed with mild ID (Intelligence Quotient = 70) and poor language abilities but had learned to read and write. Brain magnetic resonance imaging was normal. No other abnormalities were observed.

### Family 3

This boy was the only son of healthy, nonconsanguineous parents. There was no family history of ID or other genetic disorders. The maternal siblings were apparently healthy (Fig. 1). The mother underwent an uneventful pregnancy that ended in a spontaneous vaginal delivery at 38 weeks of gestation. He had normal birth measurements: weight 3.2 kg (38.2%), length 51 cm (63.3%) and head circumference 34.5 cm (50%). No feeding difficulties were noted after birth.

His height was 94 cm (57.3%), weight 13.0 kg (32.9%) and head circumference 49 cm (46.7%) at the age of 2.5 years. Motor development was delayed: he raised his head at 6 months, sat alone at 1 year and walked independently at 2 years. He had no language development. In addition to speech delay, he showed poor motor coordination with abnormal gait and mild ID. No distinctive facial features were observed. Hypogonadism was noted, including micropenis, small testes and cryptorchidism. At 4 years 9 months, a general examination showed a height of 109 cm (46.1%), a weight of 17.8 kg (40.2%) and a head circumference of 50.5 cm (40.9%), indicating normal growth development. Cognitive evaluation was performed and demonstrated mild ID (Intelligence Quotient = 65). His language skills were still poor, and he only formed two-word sentences. Brain magnetic resonance imaging and electroencephalography were normal.

### Family 4

The patient, a 13-year-old boy, was second-born to a nonconsanguineous couple and had a healthy female sibling (Fig. 1). He was born at 37 weeks gestational age by spontaneous vaginal delivery. Birth measurements were within the normal range. Language development was significantly delayed, and he began to talk at 4 years and had simple reading skills at 11 years. He suffered from moderate ID with hypogonadism, including micropenis and small testes. He was noted to have delays in motor development: he raised his head at 7 months, sat alone at 1 year and walked at 3 years. His growth development was normal. No significant dysmorphic facial features were noted. Brain magnetic resonance imaging revealed brain dysplasia. No other abnormalities were observed.

### Whole exome sequencing (WES) and chromosomal microarray analysis (CMA)

A total 10,231 individuals with neurodevelopmental disorders were recruited to Dongguan Maternal and Child Health Care Hospital for genetic testing during 2010–2019. This study was approved by the Committee on Ethics of the Dongguan Maternal and Child Health Care Hospital. DNA of family members was extracted from peripheral blood lymphocytes using standard methods. The patients’ peripheral blood DNA was subjected to whole exome sequencing (Illumina) to screen for causal variants. The bcl2fastq2 Conversion Software (v2.20) was applied for extracting Fastq files. BWA (v0.2.10) was employed for genome alignments and variant detection. The ExomeDepth (v1.1.4) was used for CNVs analysis. Clinic Sequence Analyzer (CSA) software was used for biological analysis and interpretation. The pathogenicity of the sequence variants was interpreted in accordance with the American College of Medical Genetics and Genomics/Association for Molecular Pathology (ACMG/AMP) guidelines [11]. Chromosomal microarray analysis (CMA) was performed using the CytoScan™ HD array (Thermo Fisher). Chromosome Analysis Suite (ChAS) was applied for analysis of the raw data. The interpretation of the CNVs was performed according to the American College of Medical Genetics (ACMG) guidelines [12].

### Whole genome sequencing (WGS)

Whole genome sequencing (Illumina) was employed for the proband (Family 1) to determine the structural rearrangements and precise breakpoints of the small duplicated segment. WGS was operated with an Illumina HiSeq 2500 system (Illumina) with 150 bp pair-end reads. The target coverage was 30X, and two sequencing libraries with target insert sizes of approximately 300–400 bp were constructed and sequenced in four lanes. Agilent 2100 bioanalyzer (Agilent Technologies) was used to evaluate the quality of the sequencing libraries.

In all families, WES tests revealed no clinically significant sequence variants associated with the probands’ clinical features but implicated duplications in the Xp11.22 region. Next, CMA tests were used to confirm and refine the Xp11.22 duplications.

**Fig. 2:**
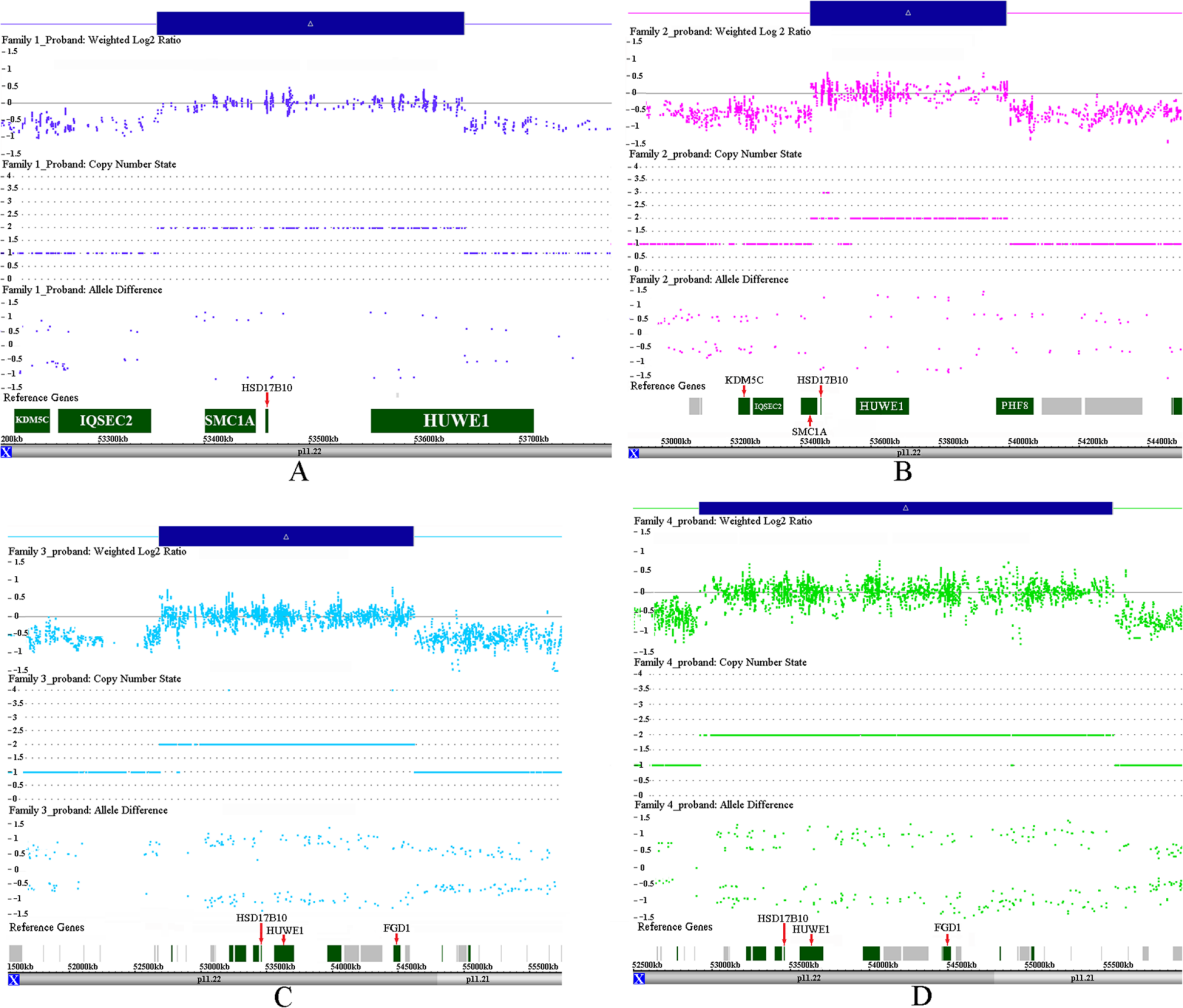
Results of CMA for the probands of four unrelated Chinese families. Affymetrix cytoscan HD analysis including weighted log2 ratio (upper), copy number state (middle) and allele difference (lower) are shown for chromosome X. The result shows duplications at Xp11.22 in the probands of four unrelated Chinese families. The duplication region is denoted by a blue bar. In family 1, a 292 kb duplication at Xp11.22 with genomic coordinates 53,355,898-53,647,498 was detected in the proband (**a**). In family 2, a 564 kb duplication at Xp11.22 with genomic coordinates 53,428,070-53,992,238 was identified in the proband (**b**). In family 3, a 1.9 Mb duplication was detected at Xp11.22 with genomic coordinates 52,686,671-54,621,465 in the proband (**c**). In family 4, a 2.6 Mb duplication at Xp11.22 was revealed with genomic coordinates 52,923,471-55,551,584 in the proband (**d**)

**Fig. 3:**
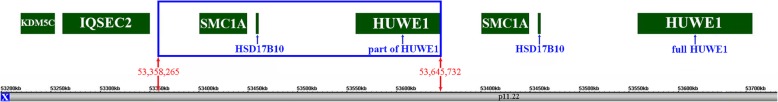
Structural rearrangement of the Xp11.22 microduplication in the proband (Family 1). WGS shows that the small duplication (Family 1) is 287 kb with precise genomic coordinates chrX:53,358,265-53,645,732 (GRCh37/hg19) spanning the entirety of the *HSD17B10* and *SMC1A* genes, as well as part of *HUWE1*, and reveals that the duplication was in tandem orientation. The duplication region is surrounded by a blue box. Obviously, *HUWE1* is partially duplicated, which suggests one functional copy of the gene

In Family 1, a 292 kb duplication at Xp11.22 was detected in the proband, the mother and the affected maternal uncle. The genomic coordinates were chrX:53,355,898-53,647,498 (GRCh37/hg19), encompassing the entirety of the *HSD17B10* and *SMC1A* genes, as well as part of *HUWE1* (Fig. 2). The proband’s healthy younger brother did not inherit the duplication from the mother. The WGS test showed that the small duplication was 287 kb with precise genomic coordinates chrX:53,358,265-53,645,732 (GRCh37/hg19) spanning the full *HSD17B10* and *SMC1A* genes, as well as part of *HUWE1*, and verified that the duplication was in tandem orientation. Apparently, *HUWE1* was partially duplicated, which suggested one functional copy of the gene (Fig. 3).

In Family 2, a 564 kb duplication at Xp11.22 was identified in the proband. The genomic coordinates were 53,428,070-53,992,238 (GRCh37/hg19) (Fig. 2). The unaffected mother was the carrier of the duplication, which was most likely an inherited event because of the remarkable family history of most males suffering from ID and speech impairments.

In Family 3, a 1.9 Mb duplication was detected at Xp11.22 with genomic coordinates 52,686,671-54,621,465 (GRCh37/hg19) in the proband (Fig. 2). The unaffected mother carried the duplication, which was a de novo event, because the duplication was absent in the maternal grandmother and grandfather.

In Family 4, a 2.6 Mb duplication at Xp11.22 was revealed with genomic coordinates 52,923,471-55,551,584 (GRCh37/hg19) in the proband, which was inherited from the unaffected mother (Fig. 2).

## Discussion and conclusions

**Fig. 4:**
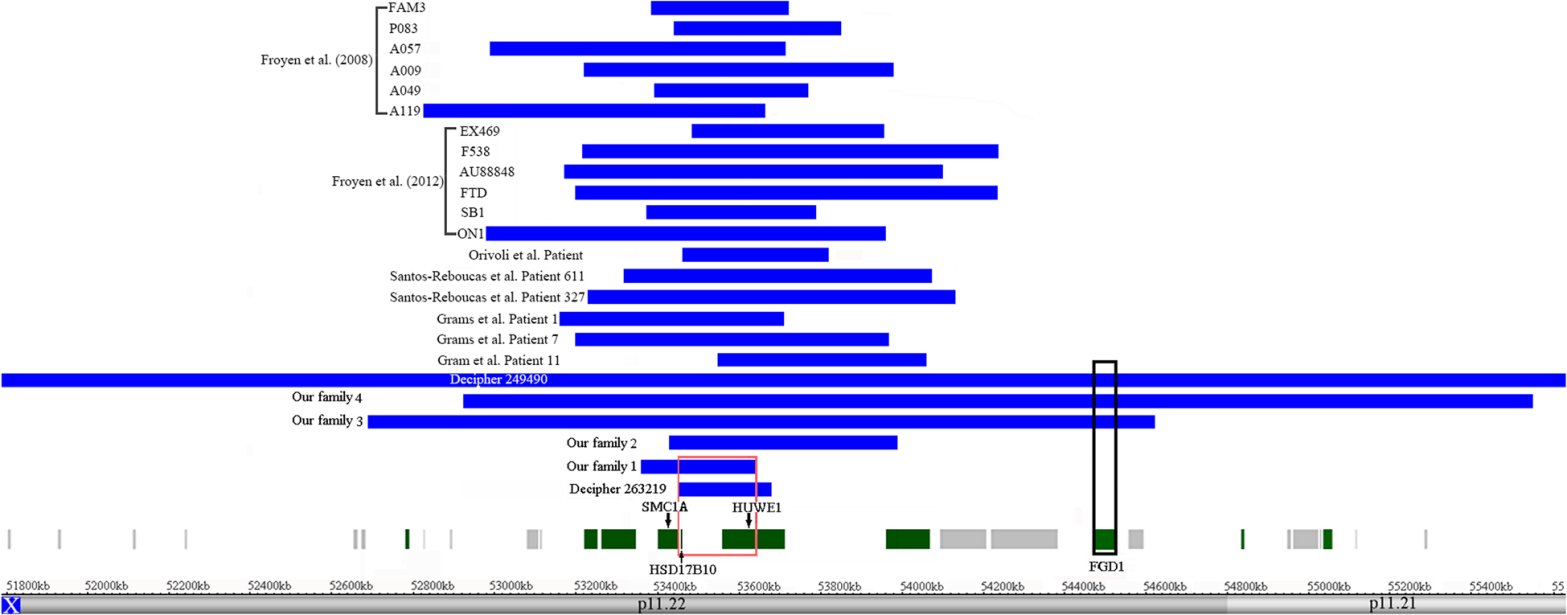
A genome view of all Xp11.22 duplication cases. The panel shows a genome view of all Xp11.22 duplication cases (blue colored custom tracks) extracted from Human Genome Build 37 (hg19). The minimal overlapping region of one of our probands (Family 1) and DECIPHER 263219 only spans the entire *HSD17B10* gene, which is surrounded by a red box. The *FGD1* gene is duplicated exclusively in two of our probands (Families 3 and 4) and DECIPHER 249490, which is surrounded by a black box

Nonrecurrent Xp11.22 duplications have been reported to be relevant to nonsyndromic ID. At present, patients with overlapping duplications at Xp11.22 have been described in 18 unrelated families. Comparing all male patients with the Xp11.22 duplication, we found that the commonly shared characteristics were the presence of a mild-to-moderate ID and language delay [6–10]. A common minimal overlapping region was identified that contained only the *HUWE1* gene. Therefore, it was concluded that *HUWE1* was likely to be a dosage-sensitive gene and that an increased dosage of *HUWE1* was believed to be responsible for nonsyndromic ID [7, 8]. To date, little is known about the clinical consequences of other genes involved in this interval. Here, we presented an additional four unrelated Chinese families with Xp11.22 duplications. All male patients demonstrated similar clinical features, including ID, speech impairments and motor delay, as the most consistent features of individuals with Xp11.22 duplication. To further establish the genotype-phenotype correlations, we searched and compared all Xp11.22 duplication cases as shown in Table 1 and Fig. 4.

**Table 1:** Clinical features observed in patients with duplications at Xp11.22. The genomic coordinates are based on GRCH37/hg19

Reference	Genomic location (chrX)	Size	Origin	ID Growth	Growth delay	Motor delay	Language delay	Facial dysmorphism	Other phenotypes
Our family 1	53,358,265-53,645,732	287 kb	mat	+	–	+	+	–	
Our family 2	53,428,070-53,992,238	564 kb	mat	+	–	+	+	–	ADHD
Our family 3	52,686,671-54,621,465	1.9 Mb	mat	+	–	+	+	–	micropenis, small testes, cryptorchidism
Our family 4	52,923,471-55,551,584	2.6 Mb	mat	+	–	+	+	–	micropenis, small testes
DECIPHER 263219	53,449,334-53,681,188	232 kb	de novo	+	+	+	+	+	
DECIPHER 249490	51,786,514-55,633,505	3.9 Mb	unknown	NR	NR	NR	+	+	micropenis, small testes
Froyen et al. (2008) FAM3 [7]	53,384,357-53,723,273	339 kb	mat	+	–	NR	+	+	ADHD
Froyen et al. (2008) P083 [7]	53,440,734-53,852,372	412 kb	unknown	+	–	NR	+	–	
Froyen et al. (2008) A057 [7]	52,987,653-53,713,244	726 kb	unknown	+	–	–	+	–	ADHD
Froyen et al. (2008) A009 [7]	53,220,275-53,981,275	761 kb	mat	+	–	NR	NR	NR	
Froyen et al. (2008) A049 [7]	53,392,906-53,770,862	378 kb	mat	+	–	NR	NR	NR	
Froyen et al. (2008) A119 [7]	52,823,215-53,664,301	841 kb	unknown	+	–	+	+	–	ADHD, seizure
Froyen et al. (2012) EX469 [8]	53,484,936-53,957,276	472 kb	mat	+	NR	NR	+	+	seizure
Froyen et al. (2012) F538 [8]	53,216,303-54,239,670	1.0 Mb	mat	+	NR	NR	+	+	
Froyen et al. (2012) AU88848 [8]	53,169,907-54,101,252	931 kb	mat	+	NR	NR	NR	NR	
Froyen et al. (2012) FTD [8]	53,198,995-54,237,527	1.0 Mb	mat	+	–	+	+	+	ADHD, cryptorchidism
Froyen et al. (2012) SB1 [8]	53,370,418-53,790,660	420 Kb	mat	+	–	+	+	+	
Froyen et al. (2012) ON1 [8]	52,977,428-53,963,113	986 Kb	mat	+	–	+	+	+	ADHD
Orivoli et al. (2016) Patient [10]	53,459,179-53,822,042	363 kb	de novo	+	+	+	+	+	
Santos-Rebouças et al. (2015) Patient 611 [9]	53,316,256-54,074,258	758 kb	mat	+	–	–	+	+	ADHD
Santos-Rebouças et al. (2015) Patient 3272 [9]	53,228,169-54,133,735	905 kb	mat	+	–	+	+	+	ADHD, seizure
Grams et al. (2016) Patient 1 [6]	53,160,114–53,713,154	897 kb	mat	+	–	+	+	+	ADHD
Grams et al. (2016) Patient 7 [6]	53,198,565–53,969,809	771 kb	mat	+	+	+	+	+	ADHD
Grams et al. (2016) Patient 11 [6]	53,548,808–54,062,110	513 kb	mat	+	+	+	+	+	

*ID* Intellectual disability, *ADHD* Attention deficit hyperactivity disorder, *NR* Not reported.

We observed that the proband (Family 1) and DECIPHER patient 263,219, who carried a small duplication at Xp11.22 partially overlapping *HUWE1* also presented with ID and language delay. The partial *HUWE1* copy number gain would probably not contribute to ID, as demonstrated in families with the partial *HUWE1* duplication previously reported, since segregation analysis provided contradictory evidence [8]. The partial *HUWE1* duplication is also reported in the Database of Genomic Variants (esv3576879). In Family 1, the duplicated segment entirely encompassed the *HSD17B10* and *SMC1A* genes. The boy suffered from ID, speech impairments and motor delay. This duplication was inherited from the unaffected mother. The proband’s duplication-negative younger brother had a normal development, and the duplication-carrying maternal uncle showed a similar clinical presentation to that of the proband. The cosegregation evidence from this family implied that the small duplication at Xp11.22 was a disease-causing effect. Next, the WGS test was used to analyze the structural rearrangements of the duplication. The small duplication was confirmed to be in tandem orientation, and *HUWE1* was partially duplicated, which suggested one functional copy of the gene [Fig. 3]. Furthermore, the WGS test also confirmed that there were no other known genetic causes responsible for the clinical features of the proband. It was found that DECIPHER case 263,219, who carried a de novo small duplication at Xp11.22, entirely spanning *HSD17B10* but only partially *HUWE1* and *SMC1A,* displayed speech impairments and global developmental delay. The de novo event implicated that the small duplication was clinically significant. Based on current evidence, it is reasonable to conclude that this small duplication is likely to be causally linked with the patients’ phenotype. The overlapping region of the two patients only fully covered the *HSD17B10* gene. Therefore, *HSD17B10* was regarded as the most promising gene responsible for the patient’s phenotype. The *HSD17B10* gene encodes human 17β-hydroxysteroid dehydrogenase type 10 (17β-HSD10), a mitochondrial multifunctional enzyme that catalyzes the oxidation of neuroactive steroids and the degradation of isoleucine. The enzyme can bind to various peptides and is required for normal mitochondrial maintenance [13]. 17β-HSD10 is expressed in brain tissue at different levels and is most abundant in the hippocampus [14]. Normal levels of 17β-HSD10 in various brain regions and other tissues are essential for human health, particularly normal cognitive competence [13, 15]. Mutations in the *HSD17B10* gene lead to 17β-HSD10 deficiency, a progressive neurodegenerative disorder with age at onset ranging from the neonatal period to early childhood, characterized by ID, deficits in language, motor delay, and alteration of mitochondrial morphology [16]. In addition, elevated 17β-HSD10 levels have been found in the hippocampi of Alzheimer’s patients and in a mouse model [14, 17, 18]. It is therefore likely that not only a reduced activity but also an increased dosage of HSD10 might interfere with normal cognition through the disturbed metabolism of neurosteroid modulators of GABA_A_ receptors [13, 18]. Based on the clinical and functional evidence for *HSD17B10* duplication, we propose that *HSD17B10* may be a dosage-sensitive gene and that *HSD17B10* duplication is likely to be causally linked with the patients’ phenotypes.

Interestingly, two of our other probands (Families 3 and 4) and DECIPHER patient 249,490 all presented with strikingly similar hypogonadism phenotypes, including micropenis, small testes and cryptorchidism, which have not been reported in any of the previously described Xp11.22 duplication patients. The *FGD1* gene was duplicated exclusively in the three patients. Currently, sufficient evidence has suggested that mutations or deletions in the *FGD1* gene cause Aarskog-Scott syndrome (AAS), also known as facio-digito-genital dysplasia, characterized by hypogonadism and other abnormalities with a broad spectrum of clinical phenotypes [19–22]. In this study, WES tests excluded the sequence variants of the *FGD1* gene and other known genetic factors associated with hypogonadism in the probands (Families 3 and 4). To date, little is known about the clinical significance of *FGD1* gene duplication (the triplosensitivity score for the *FGD1* gene is zero based on the ClinGen gene dosage sensitivity scoring protocol). Given that the *FGD1* gene was duplicated exclusively in the three unrelated patients who all displayed strikingly similar hypogonadism phenotypes and that other Xp11.22 duplication patients did not show this feature, it is reasonable to speculate that *FGD1* is likely to be a dosage-sensitive gene and that *FGD1* duplication may be responsible for hypogonadism observed in our patients.

It has been known that deletions and reciprocal duplications encompassing a dosage-sensitive gene could lead to clinical phenotypes that can be broadly classified into four general categories: mirrored (opposite effects), identical, overlapping, and unique [23]. For example, haploinsufficiency of *MECP2* causes Rett syndrome, a severe neurodevelopmental disorder [24]. Duplications encompassing the entire *MECP2* gene are responsible for MECP2 duplication syndrome, leading to similar phenotypes characterized by intellectual disability, global developmental delay, autistic behavior, epilepsy and recurrent infections [25]. Haploinsufficiency of *NSD1* results in Sotos syndrome, recognized by pre-postnatal generalized overgrowth with advanced bone age, intellectual disability, and a typical facial appearance [26], whereas reciprocal duplications covering the entire *NSD1* gene display reverse clinical features, including delayed bone age, microcephaly and failure to thrive [27]. Recently, more dosage-sensitive genes have gradually been revealed, although the present evidence is not sufficient but is accumulating; these genes, including *ZEB2* [28] and *AHDC1* [29], deserve further study. Here, we provide the first evidence suggesting that *HSD17B10* and *FGD1* may be such dosage-sensitive genes.

We reported on four unrelated Chinese families with individuals who carried duplications at the Xp11.22 interval, and cosegregation evidence in the four pedigrees further implicated nonrecurrent Xp11.22 duplication in nonsyndromic ID. By comparing the genotype-phenotype relationships of all patients with Xp11.22 duplications, *HSD17B10* and *FGD1* were identified as potential dosage-sensitive genes responsible for the clinical presentations observed in our patients. Additional clinical cases and functional studies are needed to prove this hypothesis.

## Data Availability

The data used and/or analyzed in the present report was deposited in the NCBI BioProject database. The data is accessible via the accession number: PRJNA627852; or via the links: https://www.ncbi.nlm.nih.gov/bioproject/ PRJNA627852.

## Abbreviations

ID: Intellectual disability
FGD1: Faciogenital dysplasia 1
AAS: Aarskog-Scott syndrome
CNVs: Copy number variants
NAHR: Non-allelic homologous recombination
LCRs: Low copy repeats
ADHD: Attention deficit hyperactivity disorder
WES: Whole exome sequencing
CMA: Chromosomal microarray analysis
WGS: Whole Genome Sequencing
ACMG/AMP: American College of Medical Genetics and Genomics/Association for Molecular Pathology
